# Telencephalic regulation of the HPA axis in birds

**DOI:** 10.1016/j.ynstr.2021.100351

**Published:** 2021-06-10

**Authors:** Tom V. Smulders

**Affiliations:** Centre for Behaviour & Evolution, Biosciences Institute, Newcastle University, Newcastle Upon Tyne, UK

**Keywords:** Avian brain, Brain evolution, Stress response regulation, HPA axis, Hippocampus;amygdala, Septum, Paraventricular nucleus

## Abstract

The hypothalamo-pituitary-adrenal (HPA) axis is one of the major output systems of the vertebrate stress response. It controls the release of cortisol or corticosterone from the adrenal gland. These hormones regulate a range of processes throughout the brain and body, with the main function of mobilizing energy reserves to improve coping with a stressful situation. This axis is regulated in response to both physical (e.g., osmotic) and psychological (e.g., social) stressors. In mammals, the telencephalon plays an important role in the regulation of the HPA axis response in particular to psychological stressors, with the amygdala and part of prefrontal cortex stimulating the stress response, and the hippocampus and another part of prefrontal cortex inhibiting the response to return it to baseline. Birds also mount HPA axis responses to psychological stressors, but much less is known about the telencephalic areas that control this response. This review summarizes which telencephalic areas in birds are connected to the HPA axis and are known to respond to stressful situations. The conclusion is that the telencephalic control of the HPA axis is probably an ancient system that dates from before the split between sauropsid and synapsid reptiles, but more research is needed into the functional relationships between the brain areas reviewed in birds if we want to understand the level of this conservation.

## 1 Introduction

The hypothalamo-pituitary-adrenal (HPA) axis is an ancient and highly conserved system across all vertebrates (Bardet et al., 2008; Ericsson and Jensen, 2016; Jenkins and Porter, 2004; Kuenzel et al., 2020; Wise and Frye, 1973). Its main function is the release and regulation of glucocorticoid hormones (henceforth GCH; cortisol or corticosterone, depending on the species) from the adrenal gland (specifically the adrenal cortex in mammals) into the blood stream. One of GCH's main functions is to regulate the release of energy from stored resources (gluconeogenesis, lipolysis). Indeed, without GCH secretion, animals cannot survive (Sapölsky, 1997). GCH is secreted in a circadian pattern, with peak secretion at the start of the active period (Kalsbeek et al., 2012). Because of its function in mobilizing stored energy, the HPA axis is also activated in response to challenges to the organism's homeostasis. This prepares the body for prolonged action by stimulating the mobilization of stored energy. It also preserves energy by shutting down non-essential physiological systems (Herman et al., 2016). The activation of the HPA axis can be reactive (in response to a physical challenge) or anticipatory (a.k.a. psychological; in response to an anticipated challenge). Whereas the mechanisms that regulate the former are mostly located in the brain stem and hypothalamus, the mechanisms involved in anticipation of psychological or social challenges are mostly located in the telencephalon, at least in mammals (Herman et al., 2016).

Unlike the diencephalon, mesencephalon, and rhombencephalon of vertebrates, which have been relatively conserved throughout evolutionary history, the telencephalon has evolved to have very different morphology in different vertebrate lineages (Jarvis et al., 2005). The question therefore occurs whether HPA axis regulation by telencephalic structures is common in non-mammalian lineages, and whether this regulation is homologous (derived from a common ancestor with mammals) or whether (some of) the telencephalic regulation has evolved several times across evolutionary history. This paper explores the telencephalic regulation of the HPA axis in birds, which belong to a lineage that diverged from the lineage giving rise to mammals over 300MYA and whose telencephalon is strikingly differently organized from the mammalian telencephalon (Jarvis et al., 2005). I will review how the HPA axis is regulated by the telencephalon in birds and compare this to what we know about mammals. The regulation of the HPA axis in mammals has been reviewed relatively recently (e.g. Herman et al., 2016), so these reviews will be used as comparison.

## 2 The Paraventricular Nucleus (PVN)

Like in mammals and many other vertebrates, avian adrenal GCH (mostly corticosterone) are produced in and released from the adrenal gland upon stimulation with Adrenocorticotropic Hormone (ACTH). In birds, there is no well-defined adrenal cortex, so chromaffin cells producing adrenaline and interrenal cells producing GCH are mixed together (Kuenzel et al., 2020). ACTH is released from endocrine cells in the anterior pituitary gland (specifically from the cephalic lobe of the anterior pituitary gland; Kuenzel et al., 2020). These cells in turn need stimulation with Corticotropin Releasing Hormone (CRH) and Arginine Vasotocin (AVT) to release ACTH into the blood stream. Whereas AVT by itself stimulates the pituitary gland slightly more strongly than CRH by itself (at least in some bird species), it is the combination of the two that triggers the strongest (super-additive) response downstream in the HPA axis (for a review of the receptor mechanisms, see Kuenzel et al., 2020). AVT and CRH are released from neurons with cell bodies in the Paraventricular Nucleus (PVN) of the hypothalamus (Bons et al., 1988; Kuenzel et al., 2020). In the 1960s–1970s, there was a long debate in the literature as to the location in the avian hypothalamus that is homologous to the mammalian PVN. In the 1967 pigeon (*Columba livia*) brain atlas, no PVN is identified (Karten and Hodos, 1967), but a combination of studies identified a region containing CRH-expressing cells with axons terminating in the median eminence (Berk et al., 1982; Bons et al., 1988; Péczely and Antoni, 1984).

Like in mammals, the avian PVN contains two major cell categories: magnocellular and parvocellular. Magnocellular cells contain Arginine Vasotocin (AVT; the equivalent of mammalian Arginine Vasopressin) or Mesotocin (MT; the equivalent of mammalian oxytocin) and have axons that extend into the posterior pituitary gland (Goodson et al., 2012; Kuenzel et al., 2020; Mikami, 1986). Recently, some magnocellular AVT neurons have been described to co-express CRH as well (Kuenzel et al., 2020). One such magnocellular group was originally labelled as the nucleus Periventricularis Magnocellularis (PVM) (Karten and Hodos, 1967). Lesion of the PVM does not affect GCH titres in the blood (Bouillé and Baylé, 1973c). The parvocellular component of the PVN can contain a range of neuropeptides, including AVT, somatostatin, met-enkephalin, thyrotropin releasing hormone, and substance P, in addition to cells containing CRH (Józsa et al., 1984, 1988; Kiss et al., 1987). The CRH-containing neurons are located in the rostrodorsal part of the PVN. CRH^+^ neurons can also be found in other parts of the hypothalamus, but these do not seem to be related to the HPA axis (Ball et al., 1989; Bons et al., 1988; Józsa et al., 1984; Kuenzel et al., 2020; Richard et al., 2004). Parvocellular AVT^+^ neurons are distributed throughout the PVN, but only the AVT^+^ parvocellular neurons in the core PVN (i.e. near the CRH^+^ neurons) are the ones that are activated during acute and chronic stress, and therefore likely to be those involved in regulating the HPA axis (Goodson et al., 2012; Kuenzel et al., 2020; Montagnese et al., 2016; Nagarajan et al., 2014). A few neurons may express both AVT and CRH, although the evidence is scant (Nagarajan et al., 2014). Both neuron types have axons that release their respective peptides into the blood at the median eminence (Bons et al., 1988; Knapp and Silver, 1995; Kuenzel et al., 2020; Mikami, 1986), to be transported by the bloodstream to the anterior pituitary gland.

The CRH-containing fibres from the PVN neurons run together and cross the Posterior Medial Hypothalamus (PMH) on the way to the median eminence (Bons et al., 1988). This explains why lesions to the PMH reduce plasma GCH titres (Bouillé and Baylé, 1973c). Stimulation in this same area increases plasma GCH titres (Bouillé and Baylé, 1973a), which peak at 14 min after stimulation, and take roughly 2 h to return to baseline (Ramade et al., 1979). Recordings of multi-unit activity in the PMH (presumed now to be from the fibres of passage) show highest activity 3 h before the circadian peak in GCH titres (Bouillé and Baylé, 1976). This is consistent with the finding that the peak of CRH in blood precedes the peak in GCH by approximately 2 h in pigeons (Sato and George, 1973).

The HPA axis in birds, as in mammals, is regulated by negative feedback loops, in which increases in GCH blood titres trigger a reduction in activity in the PVN and hence a reduction in the release of CRH and AVT. The PVN contains glucocorticoid receptors (GR), which is the low-affinity receptor for glucocorticoids (Cornelius et al., 2018; Dickens et al., 2009, 2011; Hodgson et al., 2007; Krause et al., 2015; Senft et al., 2016; Shahbazi et al., 2011; Suzuki et al., 2011) and thereby detects circulating GCH levels. This receptor is only activated by the high GCH titres obtained during activation of the HPA axis, and not by normal baseline titres. The PVN does not contain the high-affinity receptor, the mineralocorticoid receptor (MR; Senft et al., 2016). Expression levels of GR can be related to stress sensitivity. For example, GR (as well as CRHR1) expression in the hypothalamus is higher in domesticated chickens (*Gallus domesticus*) than in red jungle fowl (*Gallus gallus*) (Løtvedt et al., 2017), providing stronger negative feedback and therefore damping down peak stress responses.

The avian PVN is activated during acute stress (Berk and Butler, 1981; Nagarajan et al., 2014). It responds to all different kinds of stressors, both psychological (Calisi et al., 2008; Goodson and Evans, 2004) and physiological (heat stress, infection, osmotic; Calefi et al., 2016; Jaccoby et al., 1999), and activity levels (both baseline and stress-induced) can be sensitive to season (Calisi et al., 2008). Restraint stress leads to the activation of parvocellular AVT neurons in the core PVN (Nagarajan et al., 2014). The exact cell types activated depend on the stressor (e.g. magnocellular AVT^+^ neurons respond to osmotic stress; Jaccoby et al., 1999) or behavioural context, such as copulation and male-male aggression (Xie et al., 2010). Many papers do not investigate which exact cell types are activated, but only report that they are located in the PVN. Activation of the PVN therefore does not always result in increased levels of circulating GCH (Calefi et al., 2016), depending on the cell populations activated. Like in mammals, in addition to projecting to the median eminence, the parvocellular portion of the PVN also projects centrally, targeting brainstem areas related to the autonomic nervous system, such as the Locus Coeruleus (LC), the Nucleus of the Solitary Tract (NTS), and the motor nucleus of the vagal nerve (Berk, 1987; Berk and Butler, 1981; Berk and Finkelstein, 1983; Kuenzel, 2015; Medina et al., 2017). Stimulation of the PVN (and surrounding hypothalamic areas) results in an increase in heart rate (MacDonald and Cohen, 1973).

## 3 Hypothalamic and subpallial telencephalic afferents to the PVN

The neural influences on PVN activity in mammals have been reviewed recently (Herman et al., 2016). PVN can be stimulated by areas in the brainstem (e.g. NTS, which relays information about certain homeostatic signals, and the LC, which regulates alertness). There are also inputs from other hypothalamic nuclei (e.g. the Arcuate Nucleus involved in appetite regulation) and from subpallial telencephalic areas like the Subfornical Organ (SFO; related to fluid/electrolyte balance). The stimulation due to anticipatory (i.e. psychological) stress happens most likely through disinhibition of the PVN, with the direct inhibitory input coming from the Bed Nucleus of the Stria Terminalis (BST) and several hypothalamic areas (including medial preoptic, dorsomedial, and lateral hypothalamic nuclei and the periPVN) (Herman et al., 2016). In birds, PVN also receives input from the LC, the NTS, and possibly from the Raphe Nuclei (Korf, 1984). The HPA response to osmotic stressors is regulated by putative inputs to the PVN from the Subseptal Organ (SSO; equivalent to the mammalian SFO) and the Vascular Organ of the Lamina Terminalis (OVLT), both known to be involved in osmoregulation (Atoji and Wild, 2004; Jaccoby et al., 1999; Korf, 1984; Montagnese et al., 2004). Both SSO and OVLT also respond to a psychogenic stressor (acute restraint), with some evidence that the OVLT is more involved in psychogenic stress than the SSO (Aman et al., 2016). In the following sections, I review the evidence of connections from and regulation by a number of hypothalamic and subpallial telencephalic regions in birds.

### 3.1 Lateral Hypothalamus

A prominent hypothalamic area that is known to send inhibitory input to the PVN in mammals is the Lateral Hypothalamus (LHy) (Larsen et al., 1994), an area involved in appetite and sleep regulation. However, in rats at least, only the Intermediate Hypothalamic Area projects to the CRH-expressing parvocellular component of the PVN, while the Lateral Hypothalamic Area proper does not (Larsen et al., 1994). In birds as well, LHy has reciprocal connections with the PVN and is known to have an inhibitory effect on at least some parts of the PVN (definitely the magnocellular part, and maybe some of the parvocellular part as well) (Felix and Roesch, 1984; Korf, 1984), but we currently do not know if this affects the CRH^+^ neurons or the parvocellular AVT component that release their peptides into the median eminence. We do know, however, that LHy is activated during acute and chronic restraint stress in chickens (Nagarajan et al., 2014), and not during osmotic or haemorrhagic stress (Jaccoby et al., 1999). In at least one species, the LHy has also been shown to express GR and MR (Dickens et al., 2011).

### 3.2 Lateral Bed Nucleus of the Stria Terminalis

In birds, like in mammals, the BST is subdivided into two major parts: lateral (BSTL) and medial (BSTM). BSTL was inaccurately labelled as n. Accumbens in the older avian literature (Reiner et al., 2004). Whereas the BSTM is considered part of the medial extended amygdala (MEA) and plays a role in sexually motivated behaviours, the BSTL is considered part of the central extended amygdala (CEA) and is more involved in regulating anticipatory stress responses (Martínez-García et al., 2008; Medina et al., 2017). It is an area that integrates information from many other brain areas (Bálint et al., 2011). BSTL projects directly and reciprocally to the medial hypothalamus, with anterogradely labelled fibres from the anterior BSTL in both the magnocellular and parvocellular divisions of the PVN (Atoji et al., 2006; Bálint et al., 2011), as well as to the Lateral, Retromammilary, and Posterior Hypothalamus (Bálint et al., 2011). Like in mammals, the BSTL projects reciprocally to the NTS and several other autonomic nervous system-related and catecholaminergic nuclei in the brainstem (Atoji et al., 2006; Bálint and Csillag, 2007; Bálint et al., 2011; Berk, 1987). Interestingly, the subregions of the NTS that receive projections from the BSTL are the same subregions that also receive projections from the PVN (Berk, 1987; Berk and Finkelstein, 1983), reinforcing the idea that these areas form part of the same circuit. BSTL contains mainly GABA-ergic projection neurons (Bruce et al., 2016; Real et al., 2008), as in mammals, but also CRH^+^ neurons (Ball et al., 1989; Józsa et al., 1984; Richard et al., 2004). BSTL (at least at the level of the anterior commissure) is activated by the acute stress of a brief restraint in song sparrows (*Melospiza melodia*) (Goodson and Evans, 2004) and chickens (Nagarajan et al., 2014). This activation in song sparrows mostly involves non-CRH^+^ neurons (Goodson and Evans, 2004). It also responds to osmotic and haemorrhagic stress in chickens (Jaccoby et al., 1999). BSTL, at least in songbirds, has been shown to contain GR, but not MR (Senft et al., 2016; Shahbazi et al., 2011). Electrical stimulation in the general area of the BSTL results in escape and panic behaviour (Phillips, 1964; Phillips and Youngren, 1971). It is unclear whether this has to do with activation (or indeed inhibition) of the HPA axis.

### 3.3 Medial and lateral septal nuclei

Rostral BSTL has strong reciprocal connections to the Lateral Septal Nucleus (SL) in chickens and pigeons, and receives projections from the Medial Septal Nucleus (SM) (Atoji and Wild, 2004; Bálint et al., 2011). LHy also projects to the Septum in geese (*Anser anser*) and has inhibitory effects, especially on SM. The septum in turn projects directly to LHy (Felix and Roesch, 1984). In mammals, SL may contribute to the inhibitory input onto the CRH^+^ neurons of the PVN, although, if anything, it would be indirect (Herman and Mueller, 2006; Ulrich-Lai and Herman, 2009). In birds, electrical stimulation of the SM results in suppression of baseline GCH levels, and lesions result in flattening (by increasing the troughs) of the circadian GCH rhythm. Lesions in the SL have a similar, but smaller effect (Bouillé and Baylé, 1975). Stimulation of the septum (both SL and SM) results in reduced blood pressure, which is then followed by a delayed increase in heart rate (MacDonald and Cohen, 1973). In some (more caudal) locations, stimulation can also lead to panic responses (Phillips and Youngren, 1971). Neural activity recorded in the SM follows a circadian pattern that is out of phase with the circadian pattern in the PVN, with lowest activity about 3 h before the peak in GCH blood titres (Bouillé and Baylé, 1976). Whereas the lesion result could be due to fibres of passage from the Hippocampal Formation (HF; see below) to the hypothalamus, the return to higher firing activity after the lowest point of the day was slower in the SM than in the HF, suggesting that the recordings were from septal neurons, and not fibres of passage. This is also consistent with the fact that SM is known to express MR in several species (Cornelius et al., 2018; Krause et al., 2015; Senft et al., 2016), while SL expresses both GR and MR (Cornelius et al., 2018; Dickens et al., 2009, 2011). Both SL and SM have strong inputs to medial part of the hypothalamus (Atoji and Wild, 2004; Krayniak and Siegel, 1978; Montagnese et al., 2004), which contains the parvocellular component of the PVN (Berk et al., 1982; Puelles et al., 2009). At present, we do not know whether these projections synapse directly onto CRF^+^ or AVT^+^ parvocellular neurons in the PVN, nor if they are inhibitory (like SL projections are in mammals) or excitatory. PVN also projects back to the SL (Atoji and Wild, 2004; Berk and Finkelstein, 1983; Montagnese et al., 2008), possibly with AVT^+^ fibres (Montagnese et al., 2016). SL (but not SM) is activated during an acute (but not chronic) restraint stress in chickens (Nagarajan et al., 2014), and both are activated by acute restraint stress in song sparrows (Goodson and Evans, 2004). SM responds strongly to osmotic and haemorrhagic stress (Jaccoby et al., 1999).

### 3.4 Nucleus of the Hippocampal Commissure

SM, SL and the PVN all project to the nucleus of the Hippocampal Commissure (nHpC; formerly known as the (bed) nucleus of the Pallial Commissure (nCPa); Kuenzel et al., 2011), which sits dorsal and rostral to the SSO (Atoji and Wild, 2004; Berk and Finkelstein, 1983; Montagnese et al., 2008). Although it is discussed in the section on sub-pallial afferents to the HPA axis, it is actually thought to be pallial in origin (Abellán et al., 2010). For both septal nuclei, the connection to nHpC is reciprocal (Atoji and Wild, 2004), but there is no evidence of a direct projection from nHpC to the PVN. However, nHpC does project to the BSTL (Atoji et al., 2006), and may thus form an indirect connection to the PVN. The projection from PVN to nHpC may involve both CRH and AVT, as AVT^+^ terminals have been described in nHpC and it expresses V1aR, V1bR, CRHR1 and CRHR2 receptors (although the latter two could be for detection of locally-released CRH; Kadhim et al., 2020; Montagnese et al., 2016; Nagarajan et al., 2017a). There is currently no known mammalian equivalent of the nHpC, although the mammalian medial or triangular septal nuclei have been suggested as candidates (Kadhim et al., 2019, 2020). nHpC contains CRH^+^ neurons, which are activated in response to acute restraint stress (Ball et al., 1989; Nagarajan et al., 2014). In response to food deprivation, the CRH expression in nHpC increases initially, but then drops again after 4–8 h, while CRH in PVN increases steadily throughout this period. This indicates that nHpC may play a role in the initial activation of the HPA axis (at least in response to food deprivation) (Kadhim et al., 2019; Nagarajan et al., 2017b). Electrical stimulation in the nHpC of ducks (*Anas platyrhynchos*) results in defecation, which could be in response to an acute stress response, although no other fear behaviours were observed (Phillips, 1964). CRH expression in nHpC can be triggered by AVT (Nagarajan et al., 2017a). These CRH^+^ neurons do not seem to have axons projecting to the median eminence (Knapp and Silver, 1995), and therefore do not contribute directly to the HPA axis. nHpC expresses GR, and levels of GR expression are low when CRH expression is high, and vice versa (Kadhim et al., 2019). However, dexamethasone does not seem to be able to suppress CRH release *in vitro* (Nagarajan et al., 2017a), so the relationship between GCH, GR expression and CRH release in the nHpC is still unclear.

## 4 The role of the avian amygdala in regulating the HPA axis

### 4.1 Stimulatory influences on the HPA axis

In mammals, the main telencephalic regions involved in activating the HPA axis are part of the prefrontal cortex (the infralimbic portion of medial prefrontal cortex (mPFC) in rats) and the amygdala (Herman et al., 2016). The homologies between the avian telencephalon and mammalian cortex are very contentious at best (e.g. Pessoa et al., 2019; Stacho et al., 2020), and the potential equivalent of prefrontal cortex will be discussed briefly below. However, there are parts of the avian brain, namely in the Arcopallium (formerly known as Archistriatum; Reiner et al., 2004), that have been hypothesized to be homologous to the mammalian amygdala for a very long time (Zeier and Karten, 1971). In the 1970s, J.D. Baylé, C. Bouillé, and colleagues, based at the University of Montpellier II in France, carried out a programme of research to elucidate the role of the arcopallium in the control of the HPA axis in pigeons. They discovered that isolating the hypothalamus from external inputs did not change the HPA response to ether stress, but did dramatically change the response to a restraint stressor, and removed the circadian cycle (Bouillé et al., 1973). Removal of the entire telencephalon also removed the circadian GCH cycle. However, this did not remove the ability of the HPA axis to respond to an acute restraint stress (Baylé, 1976) or electric shock (Ramade and Baylé, 1982). Indeed, only posterior de-afferentation of the hypothalamus (and not disconnection from the thalamus) removed the response to electric shock (but not to ether stress), suggesting the response to a psychological stressor is controlled at least in part by input arriving from the mid-or hindbrain (Ramade and Baylé, 1982).

Despite these results in thalamic pigeons, they showed that in intact pigeons, the telencephalon (and more specifically the arcopallium) does play a role in activating the HPA axis in response to anticipatory stressors. Electrical stimulation in the caudal ventral arcopallium (5.5 mm anterior according to Karten and Hodos, 1967), but not stimulation further rostrally (6.75 mm anterior), stimulated an increase in plasma GCH titres in pigeons (Bouillé and Baylé, 1973b). Lesioning of the same area did not affect baseline GCH levels, and stress-induced GCH levels were not investigated in that study. Stimulating the dorsal arcopallium (coordinates not given) also resulted in increased GCH levels, and lesions in that area flattened circadian GCH rhythms by suppressing peak levels (Bouillé and Baylé, 1975). Like in thalamic pigeons, cutting the input to the hypothalamus from the posterior direction in intact pigeons removed restraint-stress-induced increases in plasma GCH, while anterior deafferentation did not. Posterior deafferentation did not change circadian rhythmicity of GCH titres, while anterior deafferentation did (Bouillé et al., 1975). This indicates that the stimulatory input, responding to psychogenic stressors, is brought into the hypothalamus via a posterior route. The most parsimonious interpretation of these results is that for psychological stressors like restraint and pain (electric shock), stimulation by brain stem nuclei (like NTS and LC) is sufficient to activate the HPA axis. However, the arcopallium is necessary to stimulate the HPA axis as part of its circadian rhythm. Whether the arcopallial input is also part of the stimulation in response to psychological stressors, as would be expected from homologies with mammals, is still unclear. I will therefore explore the avian amygdala specifically for its response to stressful situations, and its connections to the PVN.

### 4.2 The avian sub-pallial amygdala

Martínez-García et al. (2008) argue that the vertebrate amygdala is strongly conserved and split into two major systems. The medial extended amygdala system, which includes the subpallial area sometimes referred to as the nucleus Taeniae (TnA; see below for discussion of structures by that name) and the BSTM as its main output to the hypothalamus, is mainly involved in socio-sexual behaviours (Abellan and Medina, 2009; Mayer et al., 2017; Medina et al., 2019; Vicario et al., 2017; Xie et al., 2010; Yamamoto et al., 2005). The central-basolateral system, on the other hand, controls fear, anxiety and stress, and includes the Subpallial Amygdala (SpA) and the BSTL, as well as several areas in the Arcopallium (including the caudal and dorsal areas referred to above (Kuenzel et al., 2011; Medina et al., 2017; Vicario et al., 2014; Vicario et al., 2015; Yamamoto et al., 2005). SpA is located ventral to the caudal Lateral Striatum and Globus Pallidus, and has direct connections to the BSTL (Atoji et al., 2006). Developmental studies have shown that this area includes distinct neuronal subpopulations which are comparable to those found in the mouse central amygdala (Vicario et al., 2014, 2015), but the role of these cells during the stress response is still unclear.

Nucleus Taeniae of the Amygdala (TnA) is part of the subpallial medial amygdala (Kuenzel et al., 2011; Medina et al., 2017; Reiner et al., 2004; Vicario et al., 2017). However, not the whole structure indicated in the pigeon atlas (Karten and Hodos, 1967) is believed to be subpallial; only the rostromedial part of this structure may be subpallial, with the caudolateral pole being part of the pallial Amygdala (Medina et al., 2017; Reiner et al., 2004; Vicario et al., 2017). In fact, the brain structure called the TnA in songbirds may not be homologous to that in pigeons and chickens, and represents a pallial aspect of the amygdala (Vicario et al., 2017). TnA in pigeons (both anterior and posterior sections) projects strongly to the BSTL (Atoji et al., 2006) and has strong reciprocal connections with SL and weaker ones with SM (Atoji and Wild, 2004; Montagnese et al., 2004, 2008). However, the pallial medial amygdalar nucleus in chickens (labelled as ATn in Puelles et al., 2009) does not seem to connect to BSTL (Hanics et al., 2017). In ring doves (*Steptopelia risorii*) and starlings (*Sturnus vulgaris*), the respective areas labelled as TnA project directly to the LHy and PMH and maybe to the paraventricular region, and receive input from BSTL and SL. In ring doves (*Streptopelia risoria*), the efferent projections come from the caudal (pallial?) portion of the TnA and travel through the Hypothalamic branch of the Occipitomesencephalic tract (HOM; Zeier and Karten, 1971). In starlings (*Sturnus vulgaris*), TnA has similar projection targets, despite it being located further medial and posterior due to the difference in brain shape in Columbidae and Passeridae (Cheng et al., 1999). TnA contains MR in many species, including ring doves, Japanese quail (*Coturnix japonica*), Bengalese finches (*Lonchura striata* var. *domestica*), zebra finches (*Taeniopygia guttata*), and budgerigars (*Melopsittacus undulatus*) (Matsunaga et al., 2011; Shahbazi et al., 2011; Suzuki et al., 2011). It also expresses GR, but not MR, in chukar (*Alectorus chukar*) (Dickens et al., 2011). It is possible that the nucleus identified in chukar is not the equivalent of that identified in the other species.

TnA can be activated during stressful events. More neurons express immediate early genes in TnA when pigeons are exposed to unpredictable foot shocks, than if those shocks are predicted by tones (or than tones alone) (Brito et al., 2019); and when black-capped chickadees (Poecile atricapilllus) are exposed to the stress of predator cues, such as calls of a predator or high-urgency alarm calls. This activation lasts for at least 7 days after repeated exposure (Zanette et al., 2019). The structures identified in these two studies are equivalent to those identified in ring doves and starlings by Cheng et al. (1999), which had similar connectivity to each other. They therefore probably represent the same area, although it is likely not to be subpallial. On the other hand, in chickens, both acute and chronic restraint stress did not change activation levels in TnA (Nagarajan et al., 2014), nor did infection or heat stress (Calefi et al., 2016); and that same structure (as well as some other regions of the arcopallium) was activated in response to the first experience of a conspecific (Mayer et al., 2017, 2019), but not to encounters with adult chickens of the same or opposite sex (Xie et al., 2010). In these chicken studies, however, the area indicated as TnA (Kuenzel and Masson, 1988) was equivalent to the area labelled as the Amygdalohippocampal Area by Puelles et al. (2009), which may be a different part of the pallial amygdala, possibly in the Ventral or Medial Arcopallium. Some of the inconsistencies across studies may therefore be due to the authors referring to different brain structures as TnA (and using different atlases).

### 4.3 The avian pallial amygdala

The avian pallial amygdala is made up in part by the Arcopallium, which is a very complex region, with many subdivisions (Herold et al., 2018; Medina et al., 2017), and possibly in part also by the caudal Nidopallium (Martínez-García et al., 2002; Martínez-García et al., 2007; Medina et al., 2019; Pessoa et al., 2019). The posterior and medial subdivisions of the Arcopallium were suggested to be limbic in nature, while the anterior and intermedial Arcopallium was hypothesized to be somatic in nature (Davies et al., 1997; Zeier and Karten, 1971). However, this general subdivision is too simplistic. Large lesions in the anterior Arcopallium have been shown to reduce fear behaviour in an open field, while lesions in the posterior Arcopallium increased the same behaviour (Saint-Dizier et al., 2009). However, both lesions included a number of arcopallial subdivisions, so the exact location of these effects is not known. For the Arcopallium, I will use the subdivisional terminology of Herold et al. (2018), which identifies several regions in the arcopallium and the adjacent sub-pallial telencephalon as being “limbic” in nature. As I explore different subdivision of the pallial amygdala, special attention will be paid to their connections with BSTL, as the main output region to the hypothalamus, as well as direct connections to the PVN.

The Medial Arcopallium (AM) is located adjacent to TnA in pigeons, ring doves and starlings and it expresses GR, at least in chukar (Dickens et al., 2011). It is clearly limbic in its receptor expression profile (Herold et al., 2018) and has projections to BSTL and to the compact part of the Posterior Arcopallium (PoAc), as well as reciprocal connections with TnA (Atoji et al., 2006; Atoji and Wild, 2009; Cheng et al., 1999). It also has direct projections to the medial hypothalamus, possibly including the PVN, through the HOM (Atoji and Wild, 2009; Dubbeldam et al., 1997). Lesions in AM resulted in degenerating terminal fields being visible in the PVN, confirming the direct connection (Phillips and Youngren, 1986). Projections of AM in starlings and ring doves are very similar to those of TnA in those same species (Cheng et al., 1999). Electrical stimulation in the AM (and TnA) can elicit both excitatory and inhibitory responses in different areas of the hypothalamus. Responses in the more lateral areas in the hypothalamus were shorter latency and larger, while responses in the medial areas of the hypothalamus were longer latency and smaller amplitude, suggesting the connection to the medial sites (including the PVN) is polysynaptic. Whether the connections to the lateral sites are monosynaptic is still unclear (Schriber, 1978). Lesions of AM (possibly including adjacent (pallial?) TnA) resulted in suppression of the expression of a fear response, like escape behaviour (Phillips, 1964), shock avoidance (Dafters, 1975), heart rate (Cohen, 1975), and fear-suppressed feeding (Dafters, 1976). Electrical stimulation in that area can elicit escape behaviour in mallards (Phillips, 1964), and (together with stimulation in the Occipitomesencephalic tract; TOM) increases heart rate and blood pressure (MacDonald and Cohen, 1973), and trigger panic responses. The latter also occurs with stimulation in the nearby SpA and TnA (Phillips and Youngren, 1971). Another study claiming to make a lesion in the AM showed the opposite effect: an increase in tonic immobility (a measure of fear). However, these lesions were further lateral than those in the other studies and may therefore have affected a different arcopallial subdivision, possibly one of the motor regions (AV or AI), and hence affecting motor responses, rather than fear/stress responses (Maser et al., 1973). Lesions in young chicks in roughly the same area resulted in a decrease in distress calls when isolated from other chicks (Phillips and Youngren, 1986). It is unclear whether this decrease is due to a decrease in fear, or a decrease in motor responses, or both. After all, the fear response necessarily includes motor responses, so the distinction may not be as dichotomous as first suggested. When American crows (*Corvus brachyrhynchos*) were exposed to a human they had learned to fear, AM and the adjacent TnA were activated. In fact, arcopallial activation was correlated with the intentness of the attention paid by the crow to the stimuli (measured as change in blinking rate) (Cross et al., 2013).

The Posterior Arcopallium (PoA) is subdivided into two parts: the basal (PoAb) and the compact (PoAc) part, which are connected to each other (Atoji et al., 2006). PoAc has reciprocal connections with AM and SpA and has strong connections with the BSTL. It also projects to SL, nHpC, and via the HOM to LHy (but not directly to the PVN) (Atoji et al., 2006; Dubbeldam et al., 1997; Kröner and Güntürkün, 1999). It also receives input from the very rostral (subpallial?) part of TnA, and sends projections to TnA as well (Atoji et al., 2006). There are reciprocal connections from PoA (although which subdivision is less clear) with SM and SL (Atoji and Wild, 2004; Montagnese et al., 2004, 2008). The Dorsal Arcopallium (AD) has properties of both premotor and limbic structures, as it expresses a “limbic” marker (Herold et al., 2018) and projects to the BSTL, SL and SM (Atoji and Wild, 2004; Hanics et al., 2017; Montagnese et al., 2008). It also receives input from SL and SM (Atoji and Wild, 2004). It is hypothesized to be equivalent to a subdivision of the basolateral amygdala in mammals, based on developmental evidence (Medina et al., 2017), and based on the fact that it expresses the same combination of serotonin receptors (Fujita et al., 2020).

### 4.4 Summary: influence of the avian amygdala on the HPA axis

It is clear from the information reviewed above that the avian amygdala, like its mammalian counterpart, is involved in responses to psychological stressors, and has adequate connections to influence the HPA axis, although a lot more basic anatomical work still needs to be done. The anatomical pathways through which different aspects of the amygdala influence the PVN seem to be mainly twofold: areas either project to the BSTL, which integrates information from other brain areas as well (see also below), and which in turn projects to the PVN; or they project directly to the PVN (or at least the surrounding hypothalamus, like LHy) via the HOM. Indeed, some areas project to both targets, but whether this represents different cell populations or axon collaterals from the same neurons is unknown. There is therefore clear potential for these areas to activate the HPA axis, as they do in mammals. However, there have been very few direct tests of this hypothesis, and there is much work left to do to understand the exact relationships between different amygdala subdivisions and the activity of the HPA axis. It should also be pointed out again that the Arcopallium is not the only part of the avian pallium suggested to be homologous to the pallial amygdala in mammals: some authors also include the caudal nidopallium into the pallial amygdala (Martínez-García et al., 2002; Martínez-García et al., 2007; Medina et al., 2019; Pessoa et al., 2019). This is discussed briefly below, in the section discussing a potential avian equivalent to prefrontal cortex.

## 5 The role of the avian Hippocampal formation in regulating the HPA axis

### 5.1 Role of the hippocampal formation in regulating the HPA axis

In mammals, both the hippocampus and the mPFC (the supralimbic portion in rodents) are known to suppress activity of the HPA axis, especially after activation by psychogenic stressors (Ulrich-Lai and Herman, 2009). To provide this negative feedback on the HPA axis, the hippocampus (as well as mPFC) widely expresses GR. Neither forebrain structure, however, seems to directly innervate the PVN. Instead, the hippocampus (more specifically, the temporal subiculum) contains a subpopulation of glutamatergic neurons which project to some subpallial and hypothalamic structures that in turn inhibit the PVN, like the SL, BST, medial Preoptic Area (mPOA), dorsomedial hypothalamus (DMH) and areas around the PVN (pPVN). Lesion of the temporal subiculum leads to extended stress responses to psychogenic stressors (Herman et al., 2016), suggesting the hippocampus plays a role in returning the HPA axis response back to baseline after an acute stressor. The hippocampus is also involved in regulating the circadian cycle of the HPA axis. Its sensitivity to changes in baseline GCH levels is regulated through the expression of MR, which the hippocampus contains at higher levels than GR (Berardelli et al., 2013).

In birds as well, electrical stimulation in the ventral subdivision (V) of the hippocampal formation (both 5.5 mm and 7 mm anterior according to Karten and Hodos, 1967), results in a decrease of 30–60% in plasma GCH titres (Bouillé and Baylé, 1973b). Lesion of this same area results in an increase in baseline GCH, in turn resulting in a flattening (to higher values) of the circadian rhythm. Restraint stress still increased GCH levels after HF lesion, but to a smaller degree than in sham birds, which the authors explained as a consequence of changed feedback due to chronically higher baseline GCH levels in lesioned birds (Bouillé and Baylé, 1973b). Deafferentation of the hypothalamus from the anterior, but not the posterior side, also eliminates the circadian GCH rhythm (by increasing baseline GCH levels during what normally would be the lower parts of the day). It does not, however, affect stress-induced GCH titres (Bouillé et al., 1975). This suggests that the inhibitory inputs activated by the HF enter the hypothalamus from the anterior end.

Recordings of multi-unit extracellular action potentials (MUA) from the ventral HF show highest activity when GCH levels are lowest, and lowest activity when GCH titres are highest in the circadian cycle. This cycle is exactly the reverse of the pattern of activity recorded in the PMH (which is presumed to be activity of fibres of passage from the PVN; Bons et al., 1988). After lesion of the HF (as well as after complete deafferentation of the hypothalamus), the circadian rhythm in hypothalamic multi-unit activity disappeared, as did the circadian rhythm in GCH titres (Bouillé and Baylé, 1978). Complete deafferentation of the hypothalamus reduced both hypothalamic MUA and GCH titres to a lower level than HF lesions (Bouillé and Baylé, 1978), suggesting that HF lesions only removed the inhibitory input, but deafferentation removed both inhibitory and excitatory inputs. Removal of both telencephalic hemispheres (therefore including HF) does not have a large effect on the GCH peak that follows brief electrical stimulation of the hypothalamus. However, in intact birds, the peak drops down more quickly to baseline between 20 and 40 min after stimulation (Ramade et al., 1979). This may represent the influence of the hippocampal feedback loop in intact animals, suppressing activity in the hypothalamus after a rise of GCH in the blood stream. Like the mammalian hippocampus, the avian HF contains high levels of both MR and GR (Cornelius et al., 2018; Dickens et al., 2009, 2011; Hodgson et al., 2007; Krause et al., 2015; Senft et al., 2016; Shahbazi et al., 2011; Suzuki et al., 2011; Zimmer and Spencer, 2014), as well as CRHR1. This latter receptor is expressed at lower levels in domesticated chickens than in ancestral red jungle fowl, while GR levels trend towards lower levels in red jungle fowl, suggesting stronger negative feedback on the HPA axis in domesticated birds (see also PVN expression earlier) (Løtvedt et al., 2017).

### 5.2 Hippocampal subdivisions

In mammals, the hippocampus is functionally subdivided into a septal pole and a temporal pole, in which the septal pole is mostly involved in cognitive function, while the temporal pole is more involved with processing emotions and controlling the stress response, as indicated by the role of the temporal subiculum outlined above (Fanselow and Dong, 2010). A similar gradient seems to exist in the avian HF, with the rostral pole being adjacent to the septum and more involved in cognitive function, and the caudal pole more involved in stress regulation (Herold et al., 2019; Payne et al., 2020; reviewed in Smulders, 2017). Indeed, exposure to stress also activates areas in the avian HF, as it does in the mammalian hippocampus. Exposure to stressful stimuli (predator calls and alarm calls) increase activation in the (caudal) HF of black-capped chickadees, both immediately, and over the next 7 days (Zanette et al., 2019). No evidence was found for HF activation in crows exposed to different types of threatening stimuli, though (Cross et al., 2013). However, in chicks that were isolated from other chicks, and emitted strong isolation calls, the lateral part of the dorsomedial (DM) area of the HF was strongly activated, all the way from the rostral to the caudal HF (Takeuchi et al., 1996). Like in mammals (Mahar et al., 2014), chronic stress reduces the amount of cell proliferation (Lormant et al., 2020) and the numbers of doublecortin-positive (DCX^+^) neurons in the HF, especially in the caudal pole of the chicken HF (Armstrong et al., 2020; Gualtieri et al., 2019). There is also less hippocampal cell proliferation in quail selected for higher emotionality (Lormant et al., 2020). DCX^+^ neurons possibly represent newly-generated neurons (Balthazart and Ball, 2014), and at least represent neurons undergoing current plasticity (Vellema et al., 2014).

### 5.3 Anatomical connections from HF to PVN

So how does the HF inhibit HPA axis activity in birds? Bons et al. (1976) showed projections from the ventral HF to the Posterior Medial Hypothalamus (PMH; both ipsi- and contra-lateral to the target HF) (confirmed by Atoji and Wild, 2004). A follow-up study using retrograde tracing confirmed that the origin of these fibres are neurons in the ventral HF (and some in the DM or dorsolateral (DL) areas), as well as neurons in the SM (Bouillé et al., 1977). However, we now know that the PMH is not the PVN, and that the effects of lesions and recordings there were due to fibres of passage. One possibility is that the hippocampal and septal terminals in the PMH form axo-axonal synapses on the PVN axons. However, to our knowledge, there is no evidence either for or against this hypothesis. In mammals, there are no direct connections from the temporal subiculum to the PVN, although there are some projections to hypothalamic areas near the PVN (e.g. pPVN; Herman et al., 2016). In birds, there do not seem to be any direct connections from the DL area of the HF to the PVN, but there may be a few from the DM area (Atoji and Wild, 2004; Szekely and Krebs, 1996), especially from the more caudal part of the HF (Herold et al., 2019) to the PVM. There may also be some projections from the parvocellular portion of the PVN (or rather the Stratum Cellulare Internum, as then named) back to the HF (Benowitz and Karten, 1976; Casini et al., 1986). However, like in mammals, most of the connectivity is probably indirect. In mammals, the SL is one potential relay nucleus for hippocampal influences on the PVN (Ulrich-Lai and Herman, 2009). In birds as well, there are strong projections from DM (and to a lesser extent from DL) to the SL, and (weaker) projections to the SM (Atoji and Wild, 2004; Herold et al., 2019).

There are two other prominent brain areas that receive input from the hippocampus and are known to send inhibitory input to the PVN in mammals: LHy and BSTL (Larsen et al., 1994; Ulrich-Lai and Herman, 2009). In birds, LHy receives strong inputs from the HF (mostly DM, but somewhat from the DL as well) and from both SM and SL (Atoji and Wild, 2004; Casini et al., 1986; Felix and Roesch, 1984). There is also evidence for (indirect) influence of LHy on HF neuronal firing (Felix and Roesch, 1984). HF, SM and SL also project to areas slightly caudal of LHy (Casini et al., 1986), possibly corresponding to the PMH projections of Bons et al. (1976) and Bouillé et al. (1977). However, if the connectivity in birds is like that in mammals, these connections may relate to other functions of the PVN, such as appetite regulation. The ventral HF in pigeons also has small but consistent projections to the BSTL, while the DM of the HF has stronger connections (confirmed by retrograde labelling). All these projections originate throughout the rostro-caudal extent of the HF (Atoji et al., 2002, 2006), arguing against the rostro-caudal functional subdivision of the avian HF. This area in the DM may be the same area that is activated during acute stress (Takeuchi et al., 1996).

In addition to both potentially modulating the PVN, the HF also connects with subdivisions of the amygdala. The DM area of the HF projects to the PoAc and PoAb from all along its rostrocaudal axis, and these areas send sparse connections back to DM and DL (Atoji et al., 2006; Atoji and Wild, 2004; Kröner and Güntürkün, 1999). DM (and to a lesser degree DL) also projects strongly (and reciprocally) to TnA and AD (although not AM) in pigeons (Atoji and Wild, 2004; Atoji et al., 2002; Casini et al., 1986; Szekely and Krebs, 1996). Again, there are very few studies of the functional interactions among these interconnected brain areas in birds, but they are very reminiscent of the limbic system of mammals.

## 6 Is there an equivalent of pre-frontal cortex regulating the avian HPA axis?

Although generally, the homology between avian and mammalian pallium is undisputed, the existence of areas homologous to different subdivisions of mammalian neocortex in birds is still strongly debated (Gedman et al., 2021; Jarvis et al., 2005). This is especially the case for prefrontal-cortex-like areas. There is good evidence that portions of the caudal nidopallium have functional similarities to prefrontal cortical areas in mammals. As pointed out earlier, there is debate, however, about whether this area most resembles prefrontal cortex, or basolateral amygdala, an area which is reciprocally connected to prefrontal cortex in mammals, and which has many similar properties (Martínez-García et al., 2002; Martínez-García et al., 2007; Medina et al., 2019; Pessoa et al., 2019). Most of this evidence about similarities is related to the cognitive role of prefrontal cortex, such as working memory (Diekamp et al., 2002; Hartmann et al., 2017; Nieder et al., 2020; von Eugen et al., 2020), and encoding of the value of reward (Dykes et al., 2018; Kalenscher et al., 2005; Koenen et al., 2013), and not to its role in regulating emotional and stress responses. The area most studied for this is the Lateral Caudal Nidopallium (NCL), which receives strong dopaminergic projections, similar to prefrontal cortex. The caudal-most aspect of NCL has projections to limbic portions of the arcopallium (like PoA), as well as BSTL (Kröner and Güntürkün, 1999). Several subdivisions of the central caudal nidopallium (NCC) are known to project to the AM (Atoji and Wild, 2009), and could therefore play a role in regulating the stress response in birds. In fact, Shanahan et al. (2013) include NCC in their Viscero-limbic circuits (together with AM, PoA, SpA, and BSTL). Exposure of American crows to a mounted predator (red-tailed hawk; *Buteo jamaicensis*) induced activation (as measured with a PET scanner) in the caudal nidopallium (Cross et al., 2013). It is unclear, however, whether this activation is related to the stress of seeing a predator, or to the evaluation of whether this presumed predator was dangerous, since exposure to a human known to be dangerous did not activate this same brain area (Cross et al., 2013). Very little research has been done with respect to the role of the caudal nidopallium in the evaluation and regulation of stress responses.

## 7 Conclusions

**Fig. 1:**
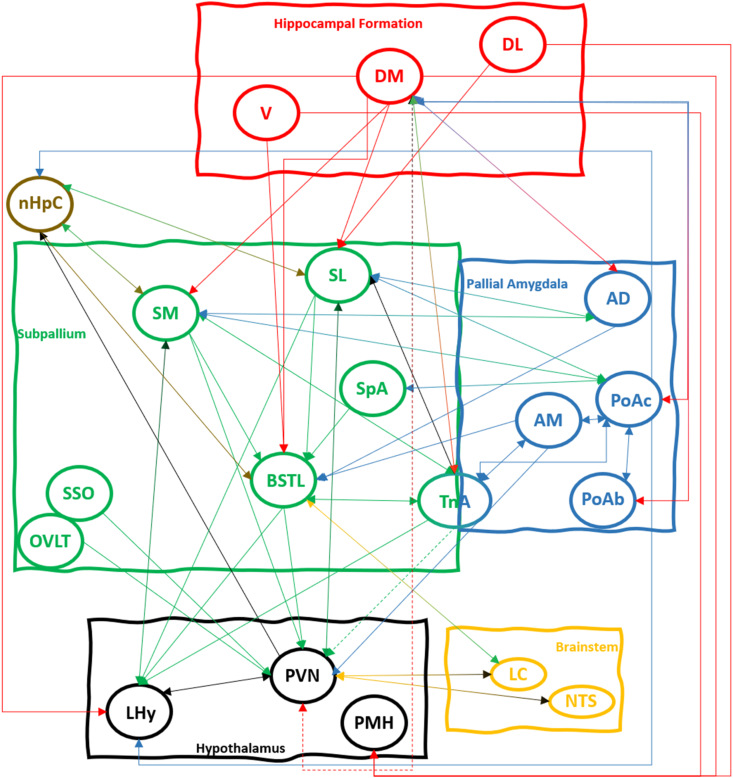
Summary diagram of all the connections between avian brain areas discussed in this review. Differently coloured boxes represent major brain subdivisions, while ovals represent individual brain areas. nHpC is a pallial structure sitting near the dorsal end of the septum (subpallium) and therefore has its own colour. Arrows represent known projections (often reciprocal) from one brain area to another. The colour of the incoming arrow indicates the origin of the arrow. The nature of the connection (whether excitatory or inhibitory) is mostly unknown and not indicated in the figure. Stippled arrows represent connections to the medial hypothalamus, but possibly not directly to PVN. For details on the connections and the references they are based on, see the main text. For abbreviations, see list of abbreviations. Note: TnA is placed in both the subpallium and the pallial amygdala, both to represent the fact that in some birds, the TnA contains different subdivisions, and that in other birds, the nucleus indicated as TnA is entirely pallial. Efferent connections from TnA have been coloured green (subpallial), unless they project to other pallial amygdala structures (blue). This is not meant to indicate that we know for sure whether those connections come from the subpallial or pallial part of TnA. (For interpretation of the references to colour in this figure legend, the reader is referred to the Web version of this article.)

The overall pattern of connectivity in the avian limbic system is quite similar to what we know about mammals. The PVN in the hypothalamus gets input from such limbic areas as the septum, the amygdala (both pallial and subpallial) and the hippocampal formation, and the few studies there are on the functional interactions between these areas are in line with what we know about mammals, with hippocampal formation and septum suppressing activity in the HPA axis and driving circadian rhythms, while the amygdala stimulates activity in the HPA axis. These general circuits suggest that the basic telencephalic influences over the HPA axis are an ancestral trait that dates back from before the split between sauropsid and synapsid reptiles, over 300 MYA. The equivalence of the influence of the medial prefrontal cortex is less clear, as there is dispute whether NCL is homologous to (at least some layers of) mPFC, or whether it is in fact an expansion of the pallial amygdala (Pessoa et al., 2019). Depending on what the situation is in reptiles, either scenario could point towards conserved circuitry between birds and mammals (Martínez-García et al., 2002; Martínez-García et al., 2007; Medina et al., 2017). Although the overall patterns of connectivity seem to have been conserved, much less is known currently about the functional relationships between the different elements of the limbic circuits summarized in Fig. 1. There is a real need for studies using modern genetic methods like optogenetics and chemogenetics to dissect out the nature of the connections (excitatory vs. inhibitory) within this network in birds and other sauropsids. Only then can we properly understand the evolution (be it conservation or divergence) of the control of the HPA axis.

## CRediT authorship contribution statement

**Tom V. Smulders:** Conceptualization, Methodology, Resources, Writing – original draft, Writing – review & editing, Visualization, Funding acquisition.

## Declaration of competing interest

The author declares that there is no conflict of interest regarding the publication of this paper.

## List of Abbreviations

ACTH –: Adrenocorticotropic Hormone
AI –: Intermediate Arcopallium
AM –: Medial Arcopallium
AV –: Ventral Arcopallium
AVP –: Arginine Vasopressin
AVT –: Arginine Vasotocin
BST –: Bed Nucleus of the Stria Terminalis
BSTL –: Lateral part of the BST
BSTM –: Medial part of the BST
CEA –: Central Extended Amygdala
CRH –: Corticotropin Releasing Hormone
CRHR –: CRH Receptor
DCX –: Doublecortin
DL –: Dorsolateral area of the Hippocampal Formation
DM –: Dorsomedial area of the Hippocampal Formation
DMH –: Dorsomedial Hypothalamus
GCH –: Glucocorticoid Hormone(s)
GR –: Glucocorticoid Receptor
HF –: Hippocampal Formation
HOM –: Hypothalamic branch of the Occipitomesencephalic Tract
HPA –: Hypothalamo-Pituitary-Adrenal
LC –: Locus Coeruleus
LHy –: Lateral Hypothalamus
MEA –: Medial Extended Amygdala
mPFC –: medial Prefrontal Cortex
mPOA –: medial Preoptic Area
MR –: Mineralocorticoid Receptor
MT –: Mesotocin
MUA –: Multi-unit activity
NCC –: Central Caudal Nidopallium
NCL –: Caudolateral Nidopallium
nCPa –: nucleus of the Pallial Commissure
nHpC –: nucleus of the Hippocampal Commissure
NTS –: Nucleus of the Solitary Tract
OVLT –: Vascular Organ of the Lamina Terminalis
PMH –: Posteromedial Hypothalamus
PoA –: Posterior Arcopallium
PoAb –: Basal nucleus of the Posterior Arcopallium
PoAc –: Compact nucleus of the Posterior Arcopallium
pPVN –: Peri-Paraventricular Nucleus
PVM –: Periventricular Magnocellular Nuclus
PVN –: Paraventricular Nucleus
SFO –: Subfornical Organ
SL –: Lateral Septal Nucleus
SM –: Medial Septal Nucleus
SSO –: Subseptal Organ
TnA –: Nucleus Taeniae of the Amygdala
TOM –: Occipitomesencephalic Tract
V –: Ventral area of the Hippocampal Formation
